# Chemical Imaging and Analysis of Single Nerve Cells by Secondary Ion Mass Spectrometry Imaging and Cellular Electrochemistry

**DOI:** 10.3389/fnsyn.2022.854957

**Published:** 2022-05-16

**Authors:** Alicia A. Lork, Kim L. L. Vo, Nhu T. N. Phan

**Affiliations:** Department of Chemistry and Molecular Biology, University of Gothenburg, Gothenburg, Sweden

**Keywords:** neurons, synapses, single cells, SIMS, mass spectrometry imaging, electrochemistry, NanoSIMS

## Abstract

A nerve cell is a unit of neuronal communication in the nervous system and is a heterogeneous molecular structure, which is highly mediated to accommodate cellular functions. Understanding the complex regulatory mechanisms of neural communication at the single cell level requires analytical techniques with high sensitivity, specificity, and spatial resolution. Challenging technologies for chemical imaging and analysis of nerve cells will be described in this review. Secondary ion mass spectrometry (SIMS) allows for non-targeted and targeted molecular imaging of nerve cells and synapses at subcellular resolution. Cellular electrochemistry is well-suited for quantifying the amount of reactive chemicals released from living nerve cells. These techniques will also be discussed regarding multimodal imaging approaches that have recently been shown to be advantageous for the understanding of structural and functional relationships in the nervous system. This review aims to provide an insight into the strengths, limitations, and potentials of these technologies for synaptic and neuronal analyses.

## Introduction

**FIGURE 1:**
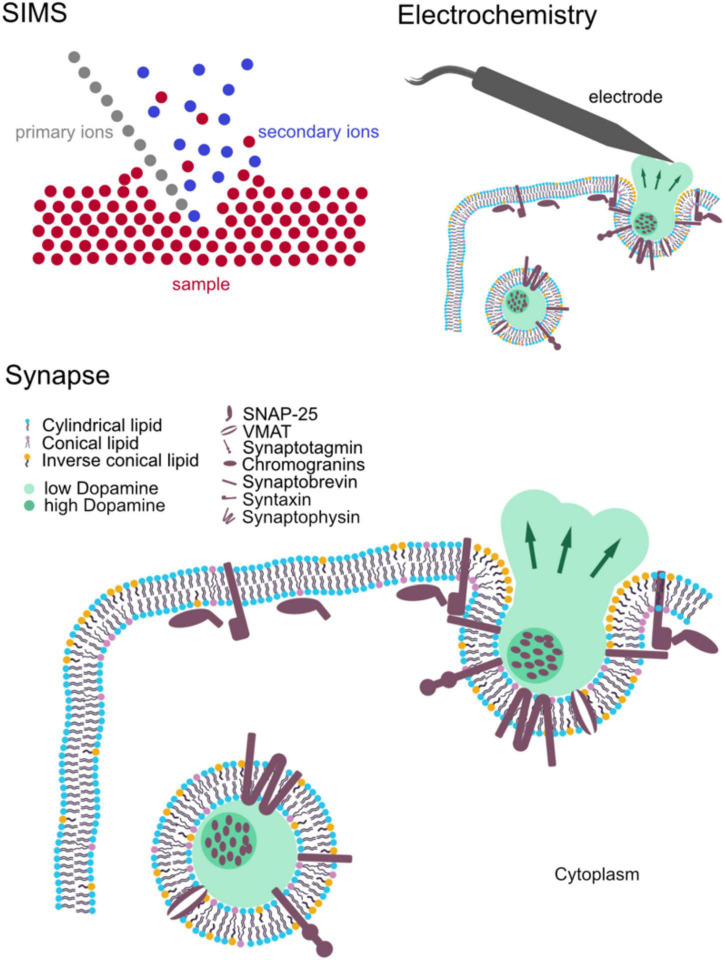
Schematic of the main components of synaptic structure: synaptic vesicles and major associated molecules affecting synaptic activity. Secondary ion mass spectrometry (SIMS) and electrochemistry for measuring synaptic structure and activity, respectively.

The molecular organization of neurons has been shown to play a regulatory role in neural communication. Investigation of this dynamic nanoscale architecture is crucially important for understanding the mechanistic insight into the brain activity and biology. Besides *in vivo* studies, neuronal cultures are widely used as models to study neuronal biology, for example, primary neuronal cultures, induced pluripotent stem cell-derived neurons, and neuronal cell lines. The main advantage of neuronal cultures is that their environmental factors, such as temperature, CO_2_ and the culture medium, can be controlled and modified in a specific manner. In addition, it is a simplified model with only one or few cell types, allowing to focus on specific cellular mechanisms or particular cellular pathways while eliminating interference from complex interactions regarding the diversity of cells in the brain. To obtain highly spatially resolved images of single synapses, spines, vesicles, etc., imaging technologies with high spatial resolution are needed. To evaluate transmitter release in single synapses, analytical techniques with high temporal resolution are desirable. These criteria can be met using presently available technologies, for instance, fluorescence microscopy and super-resolution microscopy, mass spectrometry imaging (MSI), and cellular electrochemistry. While microscopic techniques have been widely used as established methods in neuroscience and biological research, MSI and cellular electrochemistry are less known in the field (Figure 1).

Mass spectrometry imaging (MSI) is a powerful imaging tool for obtaining chemical information with cellular and subcellular spatial information. MSI is characterized by high chemical specificity, high sensitivity, high mass resolution, and, often, parallel detection of various compounds. Among different types of MSI, secondary ion mass spectrometry (SIMS) offers highest spatial resolution, which is suitable for subcellular imaging of neuronal cells. Unlike fluorescence imaging, SIMS allows for a label-free approach and elucidation of molecular structure (ToF-SIMS), although isotopic labeling is often employed to track the molecular turnover of cells and organelles (Lanekoff et al., 2011; Steinhauser et al., 2012). SIMS has continuously expanded its horizon of applications to study biomolecular organization in cell biology and neuroscience (Thomen et al., 2020; Agüi-Gonzalez et al., 2021; Jähne et al., 2021).

Electrochemical methods such as amperometry, cytometry, and voltametry measure the current or potential, which is changed by an electrochemical reaction related to an analyte, to quantify its concentration. Catecholaminergic neurotransmitters such as dopamine, epinephrine, and norepinephrine are all electroactive molecules, which can be oxidized or reduced and detected. With the excellent temporal resolution of electrochemical techniques (ns-ms range), single vesicular release events of live cells can be assessed to elucidate the mechanism of release. Electrochemical methods are, therefore, important techniques for studies on neural communication mechanisms and effects of exogeneous elements, e.g., drugs or plasticity inducing stimulation (Li et al., 2016b; Gu et al., 2019).

In this review, we present the principle, technical aspects of SIMS imaging and cellular electrochemistry, and their most relevant applications in the research on synaptic biology. The goal of this review is to introduce these unfamiliar technologies to the neuroscience community, providing an insight of their strengths, limitations, and potentials for synaptic and neuronal imaging and analysis.

## Secondary Ion Mass Spectrometry Imaging

Secondary ion mass spectrometry utilizes a primary ion beam to bombard the surface of a sample pixel by pixel, sputtering secondary ions, which originate from the sample (Pacholski and Winograd, 1999; Vickerman and Briggs, 2013). Secondary ions will then be extracted and analyzed with a mass spectrometer and eventually be detected based on their mass to charge ratio (m/z). A mass spectrum is obtained for each pixel. Assembly of all spectra corresponding to their coordinates on the surface of the sample results in ion images showing the distribution of detected ions across the sample. During bombardment of the sample surface, the energy of the primary ion beam is partially transferred to the sample surface, the so called “collision cascade” phenomenon is taking place, and molecules in the sample are sputtered and ionized. It should be noted that only a small fraction of sputtered particles is ionized and analyzed. There are a variety of primary ion sources, for example, liquid metal ion guns (LMIGs) Au_3_^+^, Bi_3_^+^, polyatomic C_60_^+^, gas cluster ion beams (GCIBs), and monoatomic Cs^+^ and O^–^. Detailed development and performance of these primary ion sources can be found in comprehensive bodies of literature (Toyoda et al., 2003; Weibel et al., 2003; Kollmer, 2004; Rabbani et al., 2013). Depending on their properties, they have different performances in terms of spatial resolution, detected ions, destruction of the sample surface, and ionization efficiency. Larger cluster ion beams are softer than the small ones (Postawa et al., 2005; Winograd, 2013 and, thus, are less destructive to the sample and generate less fragmented secondary ions. However, large ion beams have a larger beam size, which hinders lateral resolution. Furthermore, there are several other parameters that also affect secondary ion yield, particularly, ionization probability, sputter yield, and concentration of analytes, and transmission of the instrument.

### Time of Flight-Secondary Ion Mass Spectrometry and NanoSIMS

**FIGURE 2:**
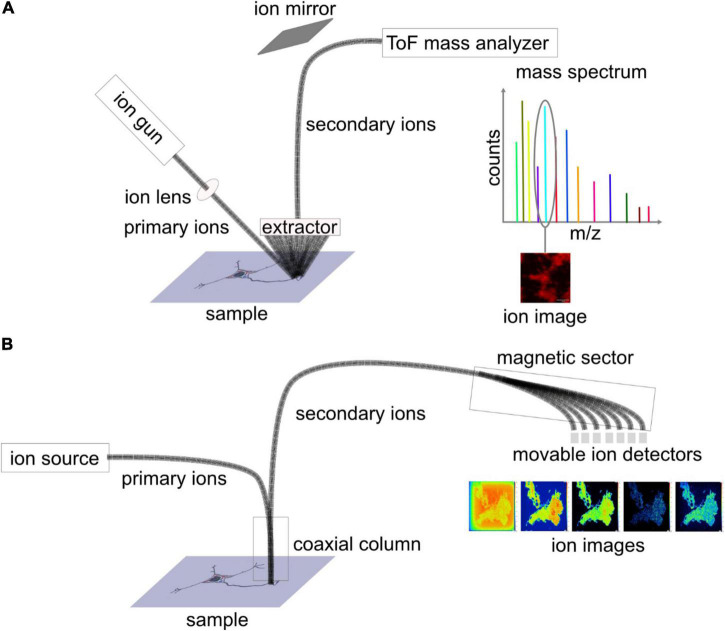
Schematic diagram depicting the set-up of **(A)** ToF-SIMS and **(B)** nanoscale SIMS (NanoSIMS). In ToF-SIMS, sample surface is bombarded with primary ion beam at an angle of 45 degrees, and secondary ions are sputtered away, extracted, and transferred into the ToF mass analyser, where the ions are separated based on their mass to charge ratio (m/z). For each pixel, a mass spectrum is obtained, and for each mass peak, an ion image is depicted. NanoSIMS can achieve higher lateral resolution owing to the coaxial design of the primary and secondary ion beams. After being sputtered from the sample surface, the secondary ions are separated by a magnetic sector analyzer, and, eventually, seven masses are detected.

There are two main types of SIMS instruments available in the field. In the following section, distinct instrumental features of these two instruments are discussed. In time of flight (ToF)-SIMS, the primary ion beam impacts the sample at a 45° angle (Figure 2A), which is one of the factors limiting achieved lateral resolutions. On the other hand, nanoscale SIMS (NanoSIMS) benefits from its coaxial design, and combined with the monoatomic primary ion beam, it provides very high lateral resolution, ∼50 nm for a Cs^+^ source (Figure 2B). SIMS generally has a depth resolution (z) of 1–10 nm. ToF-SIMS detects various analytes in parallel within a mass range of up to 2,000 Da, whereas NanoSIMS measures elements and small fragmented ions with up to 7 detectors. Mass resolution (M/ΔM) is ∼10,000 for nanoSIMS and up to 10,000 for ToF-SIMS with delayed extraction (Vanbellingen et al., 2015; Nuñez et al., 2018). In addition, NanoSIMS employs Cs^+^ and O^–^ as primary ion sources, whereas ToF-SIMS has more options from a wide range of primary ion sources mentioned above. Detailed discussion on the configuration of ToF-SIMS and NanoSIMS and their characteristics can be found in selected reviews of literature (Boxer et al., 2009; Nuñez et al., 2018; Agüi-Gonzalez et al., 2019).

### Sample Preparation for Secondary Ion Mass Spectrometry Imaging

Sample preparation is a key aspect for SIMS analysis as molecules of interest and their locations need to be preserved well. Depending on the species of interest, different strategies for sample preparation should be considered. The simplest approach in terms of sample handling is the air-drying method in which preserved cells, after multiple washing steps to remove salts etc., are air-dried. This, however, could lead to shrinkage and deformation. Distinct sample preparation protocols could be performed for particular analytes of interest because of complex chemical distribution and topography in cultured neurons (Tucker et al., 2012). Paraformaldehyde fixation retained good lipid signals of *Aplysia californica* but produced low signals in the cell soma, whereas glycerol preserved well cell mophology and retained good lipid signals in the soma. On the other hand, formaldehyde increased background signal intensity but did not enhance lipid signals. The combination of glycerol and paraformadehyde was suitable for SIMS imaging of cultured neurons. Nevertheless, chemical fixation does not work well for all types of molecules and could potentially cause interferences. To avoid this, frozen hydrated samples have been used. Samples are snap-frozen and maintained in the frozen state during measurement; this is currently the best way to preserves the sample’s molecular structure. However, it is less reproducible because of the inherent challenging procedure. One of the challenges is that samples need to be kept at a very low temperature throughout the preparation and away from the atmosphere to prevent the condensation of water on top of the samples. Another challenge is that the instrument needs to be cooled down with liquid nitrogen and maintained at equally low temperature during the entire analysis. This, in turn, causes difficulties in storing and transferring frozen samples into the analysis chamber of the SIMS instrument for analysis. A common alternative, which preserves samples well enough and is easily reproduced, is freeze-drying where samples are snap frozen in isopentane at −180°C to avoid the formation of larger water crystals. Subsequently, samples are dried in a vacuum at a low temperature so that the water slowly sublimates. Lee et al. (2019) propose covering samples with a graphene layer to let the samples dry slowly to better preserve cellular structures. The graphene layer can then be removed by air plasma treatment, and ion images can be taken with an improved quality compared to air drying. Most recently, Lim et al. (2021) showed that cells can be imaged in the wet state by covering them with grapheme, and that ions are sputtered through a transient hole while cells are still alive. Another possibility of sample preparation is to chemically fix cells with aldehydes and embed them in a resin (epoxy or acrylic resins), which has been commonly used for NanoSIMS. Samples can be contrasted, e.g., with osmium tetroxide to perform correlative transmission electron microscopy. Transparent resins can be used for correlative fluorescence microscopy (Saka et al., 2014).

Analyzing the spatial distribution of biomolecules across a biological sample has been demonstrated to be an important application for SIMS in general. Especially in highly polarized cells such as neurons, gaining the subcellular spatial distribution of cellular molecules is an important step in understanding their biology. SIMS is able to provide extensive atomic and molecular information at subcellular resolution, thus enabling the analysis of neurons, synapses, and vesicles.

### Secondary Ion Mass Spectrometry Imaging of Single Nerve Cells

#### Vitamin E in Single Neurons

**FIGURE 3:**
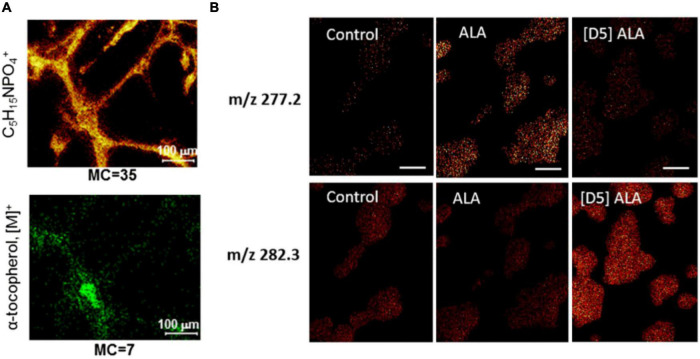
Examples of ToF-SIMS imaging in nerve cells. **(A)** Ion images of phosphocholine head group C_5_H_15_PO_4_^+^ and α-tocopherol of human iPSC-derived neurons. **(B)** Ion images of masses m/z 277.2 and m/z 282.3 of PC12 cells incubated with α-linolenic acid (ALA) and isotopically labeled D_5_-ALA. ALA-incubated cells show higher signal in m/z 277.2 than in 282.3, whereas for D_5_-ALA the signal of incubated cells in m/z 282.3 is more dominant because of the contribution of the isotopic label. The Figures were reproduced and modified from Bruinen et al. (2018) and Philipsen et al. (2018) under the creative commons license.

Many authors have studied the localization of vitamin E, an important antioxidative molecule, in neurons. The molecule is of great interest in neuroscience as low level of vitamin E affects axonal transport and can lead to axonal dystrophy and swelling. Monroe et al. (2005) identified vitamin E peaks with a vitamin E standard at m/z: 430, 205, and 165. Similar peaks were found in the *Aplysia* neuron with a distinct distribution; particularly, vitamin E localized in the soma. To minimize the effects of sample topography, the signal intensity of vitamin E was normalized to that of an ubiquitous evenly distributed ion, e.g., lipid acyl chain fragment (m/z 69). In addition, the authors highlight the importance of consistent sample preparation/analysis time as this distinct localization could not be observed when the samples were stored for a few days after freeze-drying before analysis (Monroe et al., 2005). The identification of vitamin E peaks was confirmed in *Aplysia* neurons by Passarelli and Ewing (2013) by tandem MS analysis. The localization of vitamin E was found to be in the soma, co-localizing with carotenoids, which were seen in an optical image. More recently, Bruinen et al. (2018) have imaged α-tocopherol, a type of vitamin E, in neurons (Figure 3A). Human-induced pluripotent stem cell (iPSC)-derived neurons were imaged in depth, and tandem MS analysis was performed for m/z 430. The data of MS/MS total ion current for m/z 430 as well as its product ions showed the localization of α-tocopherol predominantly in the soma but also in neurites (Bruinen et al., 2018). In addition, it was noted that peak assignments of molecules could be improved by the MS/MS information of other molecules. In this case, m/z 772 was more confidently assigned to phosphatidylcholine (PC) (32:0) [M + K]^+^ and m/z 551 as a fragment of PC (32.0) and DG (32:0). PC (32:0) [M + K]^+^ localized evenly distributed in the soma and in central axes of neurites. Interestingly, m/z 551 showed a different distribution in the outer perimeter of the soma and neurites, which is likely to be attributed to DG (32:0).

#### Organization and Turnover of Membrane Lipids in PC12 Cells

PC12 rat pheochromocytoma cells are a common, well-studied cellular model system for the investigation of exocytosis mechanisms. They are easy to maintain in a culture. PC12 cells contain large dense core vesicles (LDCVs) with catecholamines (DeLellis et al., 1973). These vesicles can release their content upon extracellular stimulation in a conserved mechanism involving multiple proteins such as SNARE proteins. This has led to the investigation of PC12 cells with different SIMS techniques. Main emphases of the studies were lipid analysis mainly with ToF-SIMS and analysis of LDCV with NanoSIMS. In the study by Lanekoff et al. (2011), PC12 cells were incubated with isotopic phospholipids of two different shapes. Incubation with cylindrical-shaped lipid PC and conical-shaped phosphatidylethanolamine (PE) was shown to alter the exocytosis rate in opposing ways (Uchiyama et al., 2007); therefore, ToF-SIMS was conducted to assess the incorporation of these lipids. The cells were incubated with lipids containing rare isotopes [deuterated 16:0/16:0 PC (D75PC) and deuterated 16:0/16:0 PE (D62PE)], which incorporated into newly synthesized lipids in cells and could be distinguished from endogenous molecules. Lanekoff et al. showed that small changes in lipids (0.5% for PC, 1.3% for PE) were observed in the plasma membrane. With increasing concentrations of up to 300 μM during incubation, the relative amount of PC and PE in the membrane also increased to ∼ 2% for PC and ∼ 9% for PE. In 2018, relative quantification of isotopic fatty acids in PC12 cells as well as their lipid turnover, a process in which old lipid molecules are gradually replaced by newly synthesized ones to maintain proper cellular functions, were investigated by Philipsen et al. (2018). A -linolenic (omega 3 fatty acid) and linoleic acids (omega 6 fatty acid) are the precursors for polyunsaturated fatty acids and lipids and have been implicated in neurological disorders (Parletta et al., 2016). Incubation of PC12 cells with α-linolenic acid and linoleic acid leads to their uptake and incorporation into PCs, PEs, and phosphatidylinositols (PIs) (Figure 3B). Interestingly, it was shown that the formation of polyunsaturated fatty acids is different depending on which isotopically labeled fatty acid was incubated. Linoleic acid was only converted to 20 carbon polyunsaturated lipids, whereas α-linolenic acid could be converted to 20 carbon and 22 carbon polyunsaturated fatty acids. Furthermore, linoleic acid and its conversion product, arachidonic acid, were found to make up lipids with two and four double bonds, while α-linolenic acid and its product, eicosapentaenoic acid, make up lipids with three, five, and six double bonds, meaning that no addition of double bonds takes place during incorporation (Philipsen et al., 2018).

#### Structural Effects of Membrane Lipids on Synaptic Plasticity

Synaptic plasticity associated with lipid alterations has recently been investigated by Gu et al. (2020). PC12 cells were stimulated 0, 3, or 6 times with a 100-mM K^+^ solution, and lipid fragments were analyzed with an ION-TOF V instrument. The authors focused on the fragments from PCs, PEs, and PIs, as they appear in higher abundance in the plasma membrane than other lipids. Interestingly, PC level reduced with increasing stimuli, whereas PE, PI, and cholesterol levels increased. It was hypothesized that this alteration might stabilize the fusion pore of vesicles during exocytosis owing to high abundance of high curvature, such as conical (PE), inverted cone shape (PI) lipids, and intrinsically negatively curved cholesterol. This, in turn, would lead to higher fraction of vesicular content to be released after multiple stimuli (Gu et al., 2020). The data agree with the results from previous studies on patients with Alzheimer’s disease (Prasad et al., 1998), cocaine-treated flies (Philipsen et al., 2020), and cognitive-enhanced methylphenidate-treated flies (Philipsen et al., 2020), suggesting a link among dynamic lipid organization, synaptic plasticity, and memory.

Agüi-Gonzalez et al. (2021) investigated the effect of neuronal activity alterations on the lipid organization of the plasma membrane in primary hippocampal neurons. First, the spatial organization of lipids and lipid fragments across different parts of the cellular membrane was assessed. Independent component analysis and neighborhood cross correlation coefficient analysis was conducted to differentiate differences in lipid localization between neurites and the cell body. Second, neurons were treated with tetrodotoxin, a voltage-gated sodium channel blocker, to inhibit neuronal activity, or with bicuculine, a competitive antagonist of GABA_*A*_ receptors, to increase neuronal activity. 51 lipid related peaks were found significantly different in their relative abundance between the activity inhibited cells and the control, and 41 lipid related peaks changed compared between the activity stimulated cells and the control. For example, ceramides, which are second messengers and components in the sphingolipid metabolic pathway, increased their abundance in inhibited cells. In addition, the total PC level decreased in the cell body while it increased in neurites in both activity-inhibited and enhanced cells. Furthermore, phosphatidylserine abundance reduced in low-activity cells, whereas it increased in high-activity ones. These distinct lipid alterations provide new insights into possible mechanisms of activity-dependent molecular organization in neurons.

## Correlative Secondary Ion Mass Spectrometry and Other Techniques for Imaging Single Vesicles and Synapses

Recently, emerging correlative imaging has expanded the perspectives of many complex biological questions that need multidimensional information, which cannot be achieved with a single technique. Correlative imaging combines several different imaging and analysis technologies to connect different properties of cells, e.g., cellular structures, functions, and molecular dynamics to obtain an insight into their molecular mechanistic process. Various combinations such as SIMS, electron microscopy, and fluorescence microscopy have been optimized (Watanabe et al., 2011; Wilhelm et al., 2014; Salamifar and Lai, 2015; Lange et al., 2021). Here, we describe several correlative approaches, which have been applied for imaging single vesicles and synapses.

### Correlative NanoSIMS and Fluorescence Microscopy

Correlative NanoSIMS and super resolution-stimulated emission depletion (STED) microscopic imaging was developed to study protein turnover in specific subcellular structures of rat hippocampal neurons (Saka et al., 2014). Neurons were incubated with ^15^*N*-leucine, which was then incorporated into newly synthesized proteins as a marker for protein turnover in neurons. Several organelles and structures, particularly active zones, vesicles, mitochondria, Golgi, and endoplasmic reticulum, were immunostained and visualized by STED microscopy. Correlation between the map of protein turnover from NanoSIMS and protein localization from STED microscopy showed that the protein turnover in the synapse is significantly higher than that in axonal regions.

The combination of isotopic ^15^*N*-leucine labeling and immunostaining for specific vesicular proteins in rat hippocampal neurons was used to study the protein turnover of recycling vesicles by NanoSIMS and STED microscopy (Truckenbrodt et al., 2018). By labeling recycling vesicles with an antibody against the intravesicular domain of synaptotagmins in different endocytosis cycles, the protein turnover of actively recycling vesicles could be compared to that of older vesicles. It was found that actively recycling vesicles are more enriched at the isotopic label and, therefore, have more newly produced proteins.

**FIGURE 4:**
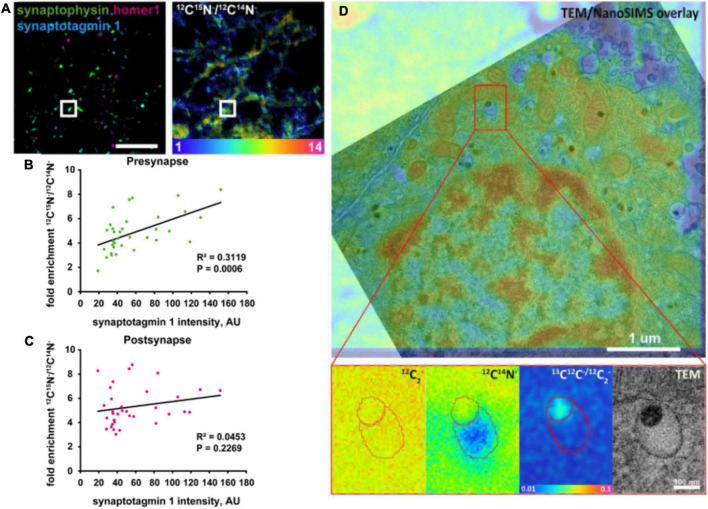
Examples of correlative imaging with NanoSIMS in neurons and synapses. **(A–C)** Correlation of synaptic protein turnover with synaptic activity. **(A)** Stimulated emission depletion (STED) images localizing the pre- and post-synapses identified by presynaptic marker (Synaptophysin), postsynaptic marker (Homer 1), synaptic activity marker (Synaptotagmin 1), and their corresponding protein turnover shown in the ^12^C^15^N/^12^C^14^N NanoSIMS image. Scale bar is 5 μm. **(B)** Quantitative chart showing the correlation of presynaptic protein turnover with synaptic activity. **(C)** Quantitative chart showing no correlation of postsynaptic protein turnover with synaptic activity. **(D)** Correlative TEM and NanoSIMS imaging of dense core vesicles in a PC12 cell incubated with ^13^C-labeled L-DOPA. A higher concentration of ^13^C-dopamine (87.5 mM) was found in the dense core compared to the halo (16 mM). The Figures were reproduced from Jähne et al. (2021) and Rabasco et al. (2022) under the creative commons license.

Correlative NanoSIMS and STED imaging was also performed to investigate protein turnover in single synapses with respect to synaptic activity in rat hippocampal neurons (Jähne et al., 2021). It was found that protein turnover is well-correlated with synaptic activity at the single synapse level. Combined with mathematical modeling of the synaptic vesicle cycle, the authors confirmed this observation by identifying the localization of synapses by STED microscopy and assessing their protein turnover by NanoSIMS using ^15^N-labeled leucine incubation technique. Pre- and post-synapses were marked with synaptophysin1 and Homer1, respectively. The intensity of both markers was not correlated with protein turnover; however, when examining only vesicles undergoing exo- and endocytosis, their synaptotagmin intensity correlated strongly with presynaptic protein turnover (Figures 4A–C). Interestingly, this correlation was abolished when the synaptic activity was altered by tetrodotoxin and bicuculine. Both drugs had an opposite effect on both pre- and postsynaptic protein turnover, which indicates that synaptic protein turnover closely depends on synaptic activity (Jähne et al., 2021).

### Correlative NanoSIMS and Electron Microscopy

The content of LDCVs has been evaluated by NanoSIMS imaging (Lovrić et al., 2017; Thomen et al., 2020; Nguyen et al., 2022; Rabasco et al., 2022). Lovrić et al. investigated the distribution of dopamine across LDCVs by incubating PC12 cells with ^13^C-labeled L-DOPA, which is then converted to dopamine and loaded into vesicles. Correlating transmission electron microscopy (TEM) and NanoSIMS, LDCVs could be identified, and their substructures, the dense core and halo, could be distinguished. The distribution of ^13^C-labeled dopamine across these substructures could also be analyzed. In addition, reserpine, an inhibitor of the vesicular monoamine transporter (VMAT) for inhibiting the loading of dopamine into vesicles, was used in this study to modulate vesicular content. Interestingly, the results indicated that the protein-rich dense core (containing, e.g., chromogranins) traps more dopamine compared to an “expandable labile pool” of dopamine in the halo with increasing incubation time with ^13^C-L-DOPA. NanoSIMS results were additionally confirmed by electrochemical data (Lovrić et al., 2017). Thomen et al. (2020) performed an absolute quantification of ^13^C-labeled dopamine level in vesicles of PC12 cells. Importantly, the concentration of carbon in the embedding material, epoxy resin, measured *via* the ^12^C^12^C^–^ signal, was similar to that in PC12 cells including vesicles. In addition, to reach a steady state of sputtering, primary ion fluence must reach 1 × 10^17^ Cs^+^cm^–2^, which consumed a sample depth of ∼180 nm. Accumulation of dopamine in the dense core of vesicles was observed to be similar to the results from Lovrić et al. (2017). The determined concentration of ^13^C-dopamine in vesicles was ∼60 mM, which is in agreement with the electrochemical data (Thomen et al., 2020). More recently, the absolute concentration of dopamine in each of two vesicle compartments was quantified (Figure 4D; Rabasco et al., 2022). It was found that the dense core contains a dopamine concentration of 87.5 mM, which is significantly higher compared to that in the halo (16 mM). Another study confirmed partial exocytotic release in PC12 cells, which is visualized by correlative NanoSIMS and TEM (Nguyen et al., 2022). ^13^C-labeled L-DOPA was incubated in cells for visualization of ^13^C-dopamine in vesicles. In addition, ^127^I-di-*N*-desethylamiodarone was introduced to cell media during KCl stimulation of cells. Exocytosed vesicles were then identified by the co-localization of ^127^I-di-*N*-desethylamiodarone and ^13^C-labeled dopamine signals inside the vesicles in NanoSIMS images. This allowed for the direct comparison of dopamine content of vesicles between stimulated and non-stimulated cells. The result showed that the dopamine content of vesicles reduces significantly after they undergo exocytosis, which confirmed the partial release of exocytosis.

## Amperometry for Studying Neurotransmitter Release During Exocytosis

**FIGURE 5:**
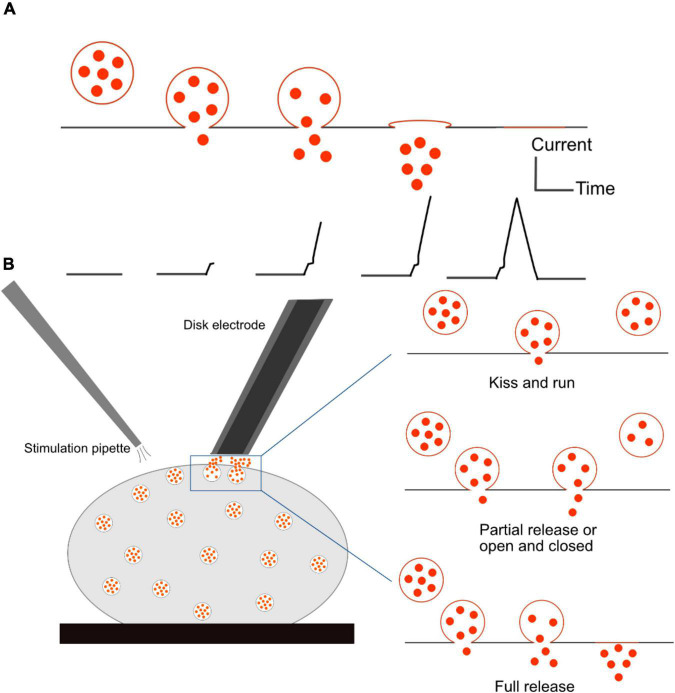
Schematic of single cell amperometry (SCA) measurement and different modes of exocytosis. **(A)** Correlation of an exocytosis event of its recorded spike. **(B)** Schematic of disk electrode placement onto a cell and a stimulation pipette to initiate exocytosis (Left), and three different modes of exocytosis: kiss and run and partial release allow for a small fraction of neurotransmitters to be released from the vesicle, while full release expels all the transmitters (Right). The fraction of released transmitter is smaller in the kiss-and-run mode than in the partial release mode.

Amperometry is one of the electrochemical methods that provides excellent sensitivity for quantification of neurotransmitters secreted from single cells at attomole to zeptomole (Chen et al., 1994; Jaffe et al., 1998; Pothos et al., 1998; Hochstetler et al., 2000), and an outstanding temporal resolution at sub-millisecond level (Robinson et al., 2008). In amperometry, a microelectrode is placed adjacent to a cell surface while a cell is stimulated with a potassium solution to initiate exocytosis. The potential is applied to an electrode surface and kept constant whereas a reduction and oxidation current will be recorded for the detection of an electroactive analyte, in this case the neurotransmitters released from the cell. A carbon fiber microelectrode was first introduced by Gonon et al. (1980) to record neurotransmitter release (Ponchon et al., 1979). In the early 1990s, single cell amperometry (SCA) was described by the Wightman group to study individual exocytosis events by placing a disk-shaped electrode on top of single bovine chromaffin cells (Leszczyszyn et al., 1991; Wightman et al., 1991). Carbon fibers have prominent mechanical and electrical properties, including high stability, long-term durability, and negligible temperature sensitivity. These electrodes can be constructed by aspirating carbon fibers (usually 5–10 μm in diameter) into glass or silica tubes to have a cylinder or a disk shaped with a 45° angle electroactive surface. Therefore, any electroactive molecules present on the surface of the electrode will react, causing a change in current (Bard and Faulkner, 2001; Borland and Michael, 2007). The recording of an amperometric peak with the presence of electroactive transmitters is shown in Figure 5A. According to Faraday’s law, the number of transmitter molecules released from each exocytosis event can be calculated with N = Q/nF, where Q is the charge in coulombs, n is the number of electrons transferred per mole in the oxidation reaction (2 electrons for the oxidation of catecholamines and serotonin), and F is Faraday’s constant (96,485 C mol^–1^). The limitation of this method is that only neurotransmitters in vesicles with accessible redox waves such as dopamine, adrenaline, noradrenaline, serotonin, and histamine can be detected. Another drawback of the technique is the lack of a qualification analysis because the applied constant potential over time cannot distinguish different electroactive analytes measured at the same time (Borland and Michael, 2007). Therefore, it is necessary to identify molecules before amperometric measurements.

### Different Modes of Exocytosis

SCA has been employed as a powerful tool to determine different modes of exocytosis. This phenomenon involves a fusion between a secretory vesicle and the plasma membrane, resulting in the release of its chemical content into the extracellular environment. The formation of a fusion pore is facilitated by SNARE proteins, but what exactly happens to the fusion pore afterward is still controversial (Ren et al., 2016). During quantal release, also called full release, a vesicle fully collapses and merges with the plasma membrane, leading to discharge of the entire content of the vesicle. The mechanism of exocytosis was thought to be an all-or-none process for a long time. However, more recent research has proved that this is not the only mode of release. There are a number of variations depending on how long the pore stays open and how much of the vesicular content is released. One of them is the kiss-and-run mechanism, in which an initial fusion pore can be transiently formed, and the vesicle releases a small amount of its content and closes. The third mode of exocytosis is referred to partial release, also known as sub-quantal release, or extended kiss and run, or open and closed. This mode involves the expansion of small sized fusion pores to a certain degree, allowing a larger amount of neurotransmitters to escape in comparison to that in the kiss and run mode. Illustrations of various modes of release are shown in Figure 5B. Flickering is another mode by which a pore opens and closes many times with only a very small fraction of catecholamine released (Staal et al., 2004). In this case, vesicles can open and close their fusion pores multiple times before expelling all the content. This phenomenon seems to appear more frequently than full release (Mellander et al., 2012).

### Amperometry for Quantifying Neurotransmitters in Single Cell Models

Owing to outstanding spatial-temporal resolution and high sensitivity, amperometry has become a powerful tool to study exocytosis for many cell types, including primary cultures, nerve cells, other secretory cell models (chromaffin cells, pheochromocytoma cells, PC12 cells, mast cells, and pancreatic β cells), neurons, synapses, and single nerve cell varicosities (Keighron et al., 2020). Quantal release was observed from axonal varicosities of midbrain dopamine neurons consisting of small synaptic vesicles by Pothos et al. (1998). A 5-μm diameter carbon electrode was placed gently onto a varicosity, which was visualized by labeling neurites with Lucifer yellow. These results provided a new mean for direct measurement of the number of neurotransmitter molecules and release duration of quantal events in central nervous system (CNS) neurons. The data also demonstrated that synaptic strength can be modulated by changing the number of dopamine molecules, or quantal size, released per exocytotic event. Zhou and Misler reported the detection of quantal catecholamine release on the surface of somas of cultured neurons by placing a carbon fiber electrode into the cleft between the somas of superior cervical ganglion neurons (Zhou and Misler, 1995). They found that 3.5 × 10^4^ catecholamine molecules were released per packet, or per quantum, indicating that release from neurons is almost 80-fold smaller than that from adrenal chromaffin cells. Another study on exocytosis events was carried out by Chen and Ewing in the cell body of single dopamine-containing neurons of *Planorbis corneus* (Chen and Ewing, 1995). Using a CNS stimulant, amphetamine, they manipulated the size and distribution of different classes of vesicles, which were distinguished by distinct distributions of the released dopamine from the neuron after the stimulation. Their results indicated a coexistence of two different classes of vesicles in dopamine containing neurons of *Planorbis*. Dopamine level was decreased by 40%, with changes in both vesicular content and distributions after amphetamine treatment. Moreover, amphetamine showed different effects on the two classes of vesicles, resulting in a third class of vesicles. The data demonstrated that multiple classes of vesicles are released, and that vesicular content can be independently manipulated with a psychostimulant. In 1995, Bruns and Jahn monitored the amount of transmitter released and kinetics of exocytosis from individual synaptic vesicles (Bruns and Jahn, 1995). The results indicated that the exocytosis of small vesicles appears more frequently and more quickly after a single action potential compared to large dense core vesicles because of different properties of their release events regarding charge, amplitude, and kinetics. Release rates of both types of vesicles are quite fast; large dense core vesicles discharge their content during the first millisecond, while small vesicles start releasing at a sub-millisecond timescale, suggesting rapid opening formation of a preassembled fusion pore. It was shown as a possible hypothesis that small vesicles are recycled without experiencing full fusion process.

### Quantifying Neurotransmitters in Single Neuronal Varicosities and Synapses

**FIGURE 6:**
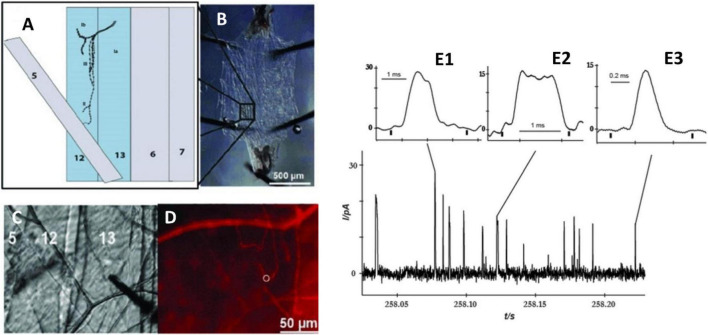
Measurement of octopamine release from *Drosophila melanogaster* larvae by amperometry. **(A)** Schematic depicting muscles from the body wall, including muscles 12 and 13, which contain type 2 octopaminergic varicosities. **(B)** Dissection of the third instar larva revealing the body muscle wall. **(C)** Position of microelectrode on type 2 varicosities in muscle 13; **(D)** Same view as panel **(C)** but under red light to visualize mCherry in octopaminergic terminals, and the white ring indicates the location of the electrode. **(E)** Different amperometric peaks of the octopamine released from varicosity stimulated by blue light. **(E1)** Two overlapping peaks, **(E2)** plateau complex event, and **(E3)** single event. This Figure was reproduced from Majdi et al. (2015) under the creative commons license.

*Drosophila melanogaster*, known as fruit fly, is an excellent model for studying brain function and neuronal communication due to its well-defined and easily manipulated nervous system. Despite its relatively simple genomes, the fly brain displays many high-order brain functions comparable to human counterparts. Therefore, *Drosophila* has been investigated to understand many important mechanisms of human developmental and physiological processes. Fly larva have been successfully developed to study the release in the field of *in vivo* electrochemistry. Majdi et al. (2015) reported a novel method to examine the octopamine release of exocytosis events in type 2 varicosities from a live, dissected larval system by amperometry. The neuromuscular junction is located peripherally and can be easily accessed with a small carbon fiber microelectrode. Furthermore, octopaminergic varicosities locate in the larval body wall, which allows an electrode to be easily positioned on these boutons from a filet of the muscle wall (Figures 6A–D). The light-activated ion channel, channelrhodopsin-2, in neuronal varicosities (type 2 optopaminergic boutons) was expressed with the m-Cherry fluorescent protein. A 5-μm carbon fiber microelectrode was positioned on top of a bouton while it was visualized under red light, and exocytosis was evoked by the activation of channelrhodopsin-2 with blue light. The number of octopamine molecules released from the varicosity was determined to be ∼22,000 per vesicle. Interestingly, different modes of release were observed based on different shapes of transients, which was considered related to the mechanism of pore opening of a vesicle to form a nanometer-wide fusion pore (Figure 6E). It was also indicated that a vesicle fusion pore just opens to release the right amount of neurotransmitters at a necessary rate. This partial release of transmitters possibly affects presynaptic plasticity.

**FIGURE 7:**
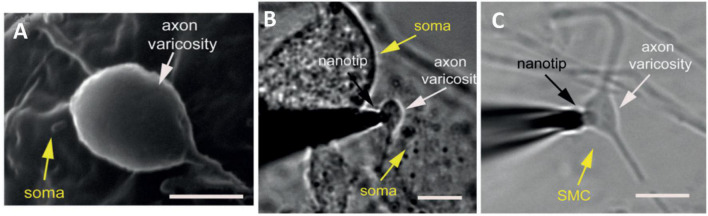
Amperometric measurements of noradrenaline release at single synapses. **(A)** Scanning electron microscope image of a synapse formed between cultured superior cervical ganglion (SCG) sympathetic neurons. **(B)** Bright field photomicrograph of the nanotip at an individual synapse, between a varicosity of one SGC neuron and the soma of another neuron. **(C)** Photomicrograph of the nanotip electrode at a synapse, between a varicosity of a SGC neuron and smooth muscle cell. These Figures were reproduced from Li et al. (2014) under the creative commons license.

Because of the small distance of a synaptic cleft between neurons (20–30 nm) (Cox and Gabbiani, 2010), it has been very difficult to measure the neurotransmitters released in an individual synapse between two communicating cells. Recently, a novel finite conical nanoelectrode with a 50- to 200-nm tip diameter was introduced for measuring individual vesicular exocytosis events at the synaptic level (Li et al., 2014). The nanotip electrode was fabricated by flame-etching a carbon fiber to form a needle-shaped electrode with less than 100-nm radius and 1-μm shaft length. The release of norepinephrine from single synapses was probed by placing the electrode inside the space between neuronal varicosities and the soma of superior cervical ganglion cells (Figure 7). The results showed two types of amperometric peaks with approximately 42% of single events and 58% of complex events. This indicated the coexistence of different types of vesicle fusions in a single synapse. Additionally, a comparison between exocytosis events inside a synapse and on top of the corresponding varicosity denoted a non-uniform distribution of active zones corresponding to these two peak types. The results suggested that neurons can adopt different vesicle fusion modes in different locations of their axonal varicosities. In another study, a quantification of dopamine release inside single dopaminergic synapses with a nanoelectrode was performed for the first time (Tang et al., 2019). Harpagide, a natural product known to be a neuroprotective drug, was unambiguously demonstrated to enhance synaptic dopamine release and restore dopamine release to normal level from damaged neurons of a Parkinson’s disease model. Phosphorylation and aggregation of α-synuclein monomers in neurons are suppressed by harpagide by inhibition of intracellular reactive oxygen level. This leads to an increase in exocytosed dopamine by increasing release frequency and total released amount, thus promoting synaptic activity.

### Biosensors for Measuring Non-electroactive Neurotransmitters in Single Neurons

Amperometric techniques with carbon fiber micro- or nano-electrodes have been commonly used to directly quantify electroactive neurotransmitters in individual exocytotic events. However, it is impossible to monitor non-electroactive neurotransmitters such as acetylcholine, glutamate (Glu), and γ-aminobutyric acid (GABA). Shen et al. (2018) investigated acetylcholine concentration and release dynamics with a 15-nm nanopipette electrode without any modifications. The pipette electrode with nanoscale tip was filled with an organic reagent and then placed in a biological environment, recordings are obtained *via* ionic fluctuations caused by ion transfer between two immiscible electrolyte solution (ITIES) interfaces. With nanoscale scanning electrochemical microscope assistance, a nanoscale ITIES can be accurately positioned at a single living *Aplysia* synapse to measure the dynamics of cholinergic transmitter release. This study revealed that acetylcholine release from *Aplysia* varicosity is Ca^2+^-dependent, and that it consists of singlet, doublet and multiplet spikes, which implies the possibility of multiple vesicle release or flickering of the fusion pore.

In another example, an enzymatic biosensor was successfully developed by co-modification of glutamate oxidase (GluOx) and platinum nanoparticles on the surface of a carbon fiber electrode by the Huang group (Qiu et al., 2018). This has been known as the first direct electrochemical detection of glutamate (Glu) released by exocytosis. The sensor was placed at a single hippocampal varicosity with stimulation of high K^+^ concentration during SCA experiments. Multiple well-defined amperometric transients of exocytosis events were obtained in the presence of GluOx on the electrode. Recently, Yang et al. (2021) developed an electrochemical Glu-nanobiosensor to measure in real-time Glu fluxes released *via* exocytosis from individual living neurons. An enzyme, GluOx, was immobilized onto a carbon-modified *SiC* nanowire to convert Glu into H_2_O_2,_ which is an electroactive molecule. The electrode was placed laterally on top of the varicosities of unpaired (without postsynaptic partner) rat hippocampal axons, and exocytosis release was triggered by a high potassium solution, leading to a series of amperometric spikes (46% occurrence frequency of single event, and the remaining had complex shapes). The study suggested a regulatory mechanism occurring during the exocytosis process to control the size, dynamics and lifetime of fusion pores, which, in turn, affects Glu release during synaptic transmission in the hippocampus.

## Vesicle Impact Electrochemical Cytometry and Intracellular Vesicle Impact Electrochemical Cytometry for Monitoring Neurotransmitter Storage in Individual Vesicles

**FIGURE 8:**
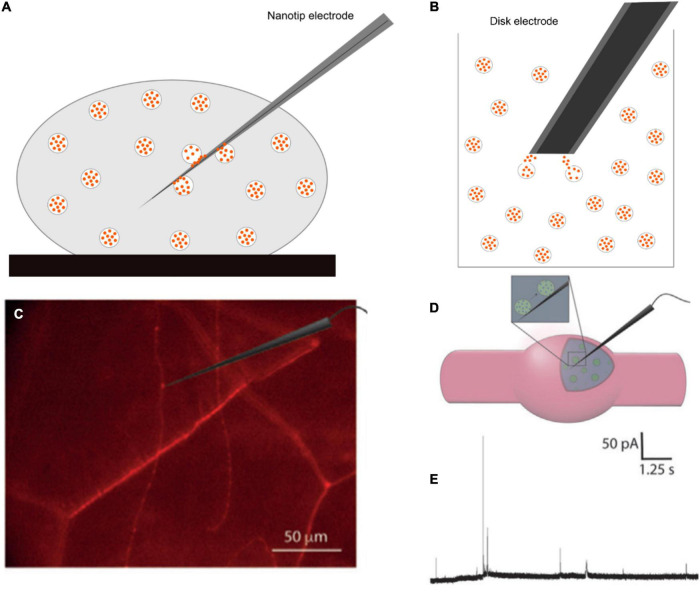
Schematic of panel **(A)** intracellular vesicle impact electrochemical cytometry (IVIEC) with a nanotip electrode, **(B)** vesicle impact electrochemical cytometry with a 33-μm disk electrode, **(C)** mCherry-labeled neurons in Drosophila larva under a fluorescence microscope and an electrode placed on or inside a varicosity, **(D)** position of a nanotip electrode in the varicosity, and **(E)** Representative current transients for IVIEC measurements. Panels **(C–E)** were reproduced from Larsson et al. (2020) under the creative commons license.

Amperometry provides an excellent method for quantification of the release process at the single cell level; however, it is unable to measure total vesicular content. This can be obtained by vesicle impact electrochemical cytometry (VIEC), and intracellular vesicle impact electrochemical cytometry (IVIEC) developed by the Ewing group (Dunevall et al., 2015; Li et al., 2016a; Figures 8A,B). These two methods share several similarities, but setups are different. In VIEC, a 33-μm disk-shaped electrode is directly dipped into a suspension of isolated vesicles. Vesicles are stochastically ruptured, and their transmitters are released to the surface of the electrode upon the application of a potential. With a similar principle, however, IVIEC employs a carbon fiber nanotip electrode (diameter < 100 nm), which allows for the electrode to penetrate into the cytosol of a living cell and measure the content of individual vesicles *in situ*. Using Faraday’s law, the number of molecules stored inside individual vesicles can be determined similarly to SCA.

The combination of SCA and IVIEC can be applied to study transmitter release and storage in secretory vesicles, and helps to unambiguously certify the quantal or sub-quantal release in different modes of exocytosis in single living cells. Fraction release, the ratio of the amount released and total vesicle content, has been shown to be affected by drug treatments (Ye et al., 2018; Zhu et al., 2019). This provides powerful methodologies allowing for the analysis of the physical properties of vesicles in order to obtain a better understanding of vesicle dynamics. Additionally, these techniques enable the understanding of how alteration of the fraction of release, under the effects of an exogenous factor, can modulate synaptic and brain activities.

Using a combination of SCA and IVIEC, Larsson et al. (2020) showed that the release of octopamine during exocytosis in *Drosophila* larval neuromuscular junction is sub-quantal and complex. In this study, a nanotip electrode was used to pierce through the muscle into octopaminergic varicosities, which were observed under a fluorescence microscope (Figures 8C–E). Total vesicle content was detected in the range of 200,000 to 1.3 million molecules per vesicle, with a median of 411,000. This value is much higher than the amount released (22,700 molecules per vesicle) previously measured by SCA (Majdi et al., 2015). The fraction release of octopamine is, therefore, very small, and accounts for 4.5% for single events and 10.7% for complex events (oscillating or flickering) compared to the total vesicle content. A fusion pore can be adjusted *via* the opening-and-closing process to increase the released amount in complex spikes compared to simple ones. Their results strongly demonstrated that presynaptic plasticity can be regulated by fluctuations of fusion pores in the partial release.

Recently, intravesicular Glu was quantified using a novel Glu biosensor in living neurons (Yang et al., 2021). A combination of SCA and IVIEC enables the determination of Glu release and intracellular storage from rat hippocampal neurons following L-glutamine and Zn^2+^ treatments, which are known to be involved in the learning and memory processes. Intravesicular contents were strongly affected in opposite ways under the two treatments. Glu vesicular content and released amount dramatically increased by 259 and 183%, respectively, under the L-glutamine treatment. However, fraction release was at a similar level compared to the control group undergoing no L-glutamine treatment. In contrast, under the Zn^2+^treatment, Glu content decreased by ca. one half, while the released amount increased by ca. one third of the total content. L-glutamine significantly enhanced the loading and synthesis of intracellular Glu but did not affect the final fusion pore, whereas Zn^2+^ tended to enlarge pore sizes. The results strengthened the concept of sub-quantal release during exocytosis.

## Other Electrochemical Methods

Another common technique is fast scan cyclic voltammetry (FSCV), which is useful for monitoring neurotransmitter fluctuations in tissues or single cells *in vitro* because of interferences of complex environments. It has been considered as the most common method for monitoring neurotransmitter level in the brain (Robinson et al., 2003, 2008; Venton and Wightman, 2003; Rodeberg et al., 2017; Roberts and Sombers, 2018). Electroactive species are quickly reduced and oxidized on electrode surface by a triangular waveform applied to the electrode at high scan rate (>100 V/s). Electroactive compounds in the CNS of a rat brain were measured for the first time using implanted graphite paste microelectrodes by the Adam group (Kissinger et al., 1973). The Wightman group successfully introduced FSCV to determine electroactive transmitters such as dopamine, norepinephrine, epinephrine, serotonin, histamine, and adenosine, from various samples, including nucleus accumbens, bovine adrenal medullary cells, mast cells, and brain tissues (Bucher and Wightman, 2015). Venton and coworkers implemented a method for monitoring dopamine release in a ventral nerve cord from Drosophila larva (Vickrey et al., 2009). They also determined extracellular serotonin and dopamine levels in a single Drosophila larva ventral nerve cord (Borue et al., 2009). ChR2 was genetically expressed in octopaminergic and serotonergic neurons, neurotransmitter released was detected by FSCV with blue light stimulation. These studies show that dopamine and serotonin regulations are analogous to those in mammals.

Chronoamperometry and differential pulse voltammetry are available for measuring concentrations of neurotransmitters and different analytes. However, these methods show limited chemical selectivity and poor time resolution, as one scan takes more than 10 s for chronoamperometry and more than 30 s for differential pulse voltammetry. The fluctuation of neurotransmitters is on the sub-second time scale; therefore, these techniques are not commonly employed for monitoring chemical communication in the nervous system.

Capacitance measurement is also a useful approach for monitoring changes of cellular surface area after the vesicular membrane has fused with the plasma membrane during exocytosis (Lindau and Neher, 1988; Gillis, 1995). It allows for direct characterization of fusion pore properties. Capacitance measurements have been conducted for cells and neurons with complex branching (Kushmerick and von Gersdorff, 2003; Kim and Von Gersdorff, 2010). Example study is whole-cell recording from mossy fiber boutons in hippocampal neurons (Hallermann et al., 2003), in axon terminals of the brainstem calyx of Held (Sun and Wu, 2001; Wölfel and Schneggenburger, 2003), and in axon terminals of neurons in the posterior pituitary gland (Hsu and Jackson, 1996). Standard capacitance measurement, however, can only measure readily releasable vesicles and, thus, is not applicable for total releasable pools.

## Summary and Future Perspectives

Measurement of molecular organization, turnover, and dynamic change in single neuronal cells and single synapses is possible using current state-of-the-art analytical technologies (Table 1). SIMS allows for the visualization of non-targeted and targeted molecules and their metabolic turnover, whereas single cell amperometry and cytometry are ideal quantification methods with high temporal resolution for neuronal secretion. These technologies offer a unique opportunity to obtain an insight into the molecular mechanism that modulates synaptic activity and neuronal processes.

**TABLE 1:** Common secondary ion mass spectrometry (SIMS) and electrochemical methods for neuronal and synaptic measurements.

Methods	Applications	Advantages/disadvantages
Time of flight secondary ion mass spectrometry (ToF-SIMS)	Imaging spatial distribution of ions up to ∼ 2,000 Da (metabolites, molecular lipids, small peptides) 2D and 3D imaging possible	+ Parallel detection within a large mass range (0-2,000 Da) +Suitable for non-targeted imaging (non-labeling) + Many primary ion sources available - Topographical sample effect - Spatial resolution possibly ∼ 250 nm
Nanoscale secondary ion mass spectrometry (NanoSIMS)	Imaging spatial distribution of monoatomic or diatomic ions at subcellular resolution 2D and 3D imaging possible	+ Spatial resolution ∼ 50 nm - Molecular information lost - Parallel detection up to 7 ions - Isotopic labeling often employed
Single cell amperometry (SCA)	Quantification of the number of neurotransmitters released from individual vesicles	+ High temporal resolution (sub-milliseconds to a few millisecond) - Cannot distinguish between catecholamine and other electroactive molecules at the same time
Vesicle impact electrochemical cytometry (VIEC)	Quantification of the total number of neurotransmitters stored inside individual vesicles Investigation of the effects of drug treatments on vesicle properties	+ easily manipulate the surrounding environment of vesicles - Risk of leakage of vesicular transmitters and changes of vesicle properties during the vesicle isolation process - Cannot distinguish between catecholamine and other electroactive molecules at the same time
Intracellular vesicle impact electrochemical cytometry (IVIEC)	*In situ* approach quantifying total vesicle content within their cellular environment (in the cytoplasm)	+ Possible to change external factors such as osmolarity, pH and pharmaceutical treatments with minimal impact on the cells - Cannot distinguish between catecholamine and other electroactive molecules at the same time
Fast scan cyclic voltammetry (FSCV)	Study of the behavior, addiction, and disease of live animals by measuring *in vivo* the rapid changes of neurotransmitters	+ Possible to simultaneously quantify and identify various analytes by selecting voltage limits of the interested analyte - Cannot measure basal levels of neurotransmitters, governed by phasic and tonic neuronal activity, only fast change of electroactive species because the background current can be only stable for a brief time

Challenges in neuronal and synaptic imaging are the required very high spatial resolution to visualize extremely small structures, sufficient sensitivity to detect analytes in a very small volume, and a suitable sample preparation strategy to preserve the intact molecular architecture of the sample. Technical developments in these technologies are still continuously ongoing to circumvent these challenges. For example, new primary ion sources and ion optics in SIMS are being developed to improve imaging sensitivity, spatial resolution, and mass resolution. Tandem MS SIMS, such as the recently developed OrbiSIMS (Passarelli et al., 2017), will be useful for elucidating molecular structures in complex molecular compositions of neurons and synapses. In addition, development of labeling tools for large biomolecules, such as proteins (Kabatas et al., 2019; Agüi-Gonzalez et al., 2021), will allow for targeted imaging of multiple proteins and lipids by SIMS at the single synapse level. Furthermore, standardization of data treatment and statistical analysis, as well as development of mass peak databases specific for neurons and synapses will ensure reliable data handling and interpretation performed by those who are not SIMS experts. This will play a vital role in realizing SIMS as a common imaging technique in neuroscience.

The development of new types of micro- and nanoelectrodes for electrochemical analysis is in continuous progress to expand its repertoire of detectable analytes, improve sensitivity and speed, and enable sub-vesicular measurements. Modification of electrochemical conditions, for example adding chaotropic anions (SCN^–^) (He and Ewing, 2022), could change the opening nature of vesicles, facilitating the measurement of transmitter content in individual sub-vesicular compartments, the halo, and dense core.

Finally, a combination of different imaging and analysis techniques to obtain multidimensional information of the studied samples will be an increasingly favored trend. We will be able to correlate the organization of a wide range of targeted molecules, neuronal structures and morphology, dynamic metabolic processes, and synaptic activity in single neurons and synapses.

## Author Contributions

AL, KV, and NP wrote the manuscript. NP organized the manuscript, coordinated, and supervised the writing progress. All authors provided comments and refined the manuscript before the submission.

## Conflict of Interest

The authors declare that the research was conducted in the absence of any commercial or financial relationships that could be construed as a potential conflict of interest. The handling editor SR declared a past co-authorship/collaboration with the author NP.

## Publisher’s Note

All claims expressed in this article are solely those of the authors and do not necessarily represent those of their affiliated organizations, or those of the publisher, the editors and the reviewers. Any product that may be evaluated in this article, or claim that may be made by its manufacturer, is not guaranteed or endorsed by the publisher.
